# Community pharmacists’ role towards preventing abuse or misuse and dependence of codeine-containing analgesic medications in Saudi Arabia: a multicenter cross-sectional study

**DOI:** 10.3389/fphar.2024.1408024

**Published:** 2024-10-14

**Authors:** Muteb Alanazi, Mukhtar Ansari, Tareq Nafea Alharby

**Affiliations:** Department of Clinical Pharmacy, College of Pharmacy, University of Hail, Hail, Saudi Arabia

**Keywords:** codeine analgesics, community pharmacists, dependence, misuse and abuse, role, Saudi Arabia

## Abstract

**Introduction:**

The misuse or abuse and dependence of medications containing codeine continue to be a major global public health concern. This study aimed to investigate the role of community pharmacists in preventing the abuse or misuse and dependence of codeine-containing analgesic drugs in Saudi Arabia.

**Methods:**

A cross-sectional study involving 226 community pharmacists from various community pharmacies across multiple cities of Saudi Arabia was conducted from 09 May 2023 to 09 October 2023. Study data were collected and managed using Research Electronic Data Capture tool. Fourteen trained data collectors visited randomly selected community pharmacies in different cities, provided pharmacists with an electronic questionnaire link, and collected their responses electronically. The dataset was downloaded in SPSS format, and analyzed for both descriptive and inferential purposes.

**Results:**

The primary indicators that community pharmacists considered when suspecting cases of abuse or misuse and dependence included customers who requested a larger quantity of the medication (88.5%), frequently visited the pharmacy and sought the specific medicine (82.7%), and those who took advantage of the crowd (70.8%). Pharmacists have been instrumental in preventing abuse or misuse and dependence by ensuring that medicines with abuse potential are not easily accessible to consumers (87.6%), providing alternative options (81%), reducing the dose (65%), referring patients to physicians (62.4%), and refusing to sell or denying availability (54.9%). Additionally, pharmacists primarily focused on raising public awareness (85%) as a noteworthy proportion of customers (54.9%) became desperate to obtain the drug after missing a dose. It was also suggested that community pharmacists should receive specialized training in substance abuse or misuse, as 46.9% of them lacked such training.

**Discussion:**

Community pharmacists, being frequently the initial point of contact easily reachable, possess the capability to greatly assist in identifying the patients and averting abuse or misuse and dependence during the dispensing of medication. Further, they can provide valuable guidance to those involved in efforts to reduce drug abuse or misuse and dependence.

## 1 Introduction

A persistent global public health issue related to the medications containing codeine is their misuse or abuse and dependence associated with them. This is especially concerning as codeine is found in both prescription and over-the-counter pain relievers, as well as cough suppressants (Van Hout and Norman, 2016). Despite being considered a mild opiate, codeine still poses risks of dependence, abuse, and misuse due to the rapid development of tolerance with frequent or excessive usage. Prolonged use of codeine-containing medications can result in the development of physical tolerance, leading to unpleasant withdrawal symptoms upon discontinuation of the medication, along with side effects such as drowsiness, euphoria, and constipation (Cooper, 2013; Nielsen and Van Hout, 2017). Codeine is often abused or misused in combination products, with consumers mistakenly believing they are taking lower dosages of the medication. However, over time, these combination products have been linked to serious clinical complications like gastrointestinal bleeding and severe hypokalemia (Mill et al., 2018). As a result, there is a growing need for increased surveillance, better detection of misuse in clinical setting, and public health awareness initiatives (Casati et al., 2012; Carney et al., 2018).

Although the pattern of misuse or abuse of codeine-containing analgesics vary from country to county, 19.5% of participants in a French study reported using codeine analgesics on a daily basis for longer than 6 months (Roussin et al., 2013). This highlights the concerning trend of codeine misuse. Similarly, studies conducted in the Middle East have shown a widespread abuse and misuse of codeine-containing medicines, often through self-medication (Casati et al., 2012; Lo et al., 2015; Khalifeh et al., 2017; Carney et al., 2018). A study by Syed et al. focused on physicians’ perspective in Saudi Arabia and highlighted the misuse of codeine-based medications (Syed et al., 2021). Another study by Yasmeen et al. in Saudi Arabia also drew attention to instances of inappropriate medicine use, including codeine-based preparations (Yasmeen et al., 2023). A substantial usage of analgesics among medical students was also noted in a study by Ibrahim et al. (Ibrahim et al., 2015). However, it is worth noting that there is a lack of research on the role of community pharmacists in preventing codeine-containing analgesic medicine abuse or misuse and dependence.

According to a survey conducted in the Eastern Region of Saudi Arabia, community pharmacists consider medication abuse and misuse to be a concerning issue (Alshayban et al., 2020). Community pharmacists in Al-Baha region of Saudi Arabia also believe that over-the-counter (OTC) medications that include codeine are among the most frequently abused and misused (Alqarni et al., 2022). Likewise, a multicenter study conducted among the community pharmacists in Saudi Arabia also raised the concern about the improper use of drugs, particularly opioids (Yasmeen et al., 2023). Additionally, a comprehensive review conducted in the Middle East region also brought attention to the misuse of drugs containing codeine, especially when self-medication is employed (Khalifeh et al., 2017). As per an additional research, community pharmacists think that patient counseling on opioid-containing medicines in particular is lacking in community pharmacy settings (Ali et al., 2023).

Given the presence of various medications containing codeine in the Kingdom of Saudi Arabia (Syed et al., 2021), we hypothesize that adolescents and young adults, who represent the future of a nation, may purposefully use these medications to elevate their mood or for recreational purposes (Kimergård et al., 2017). Additionally, they may be unaware of the addictive nature of such medications. The easy accessibility and prevalence, coupled with the covert nature of codeine-based medication abuse or misuse and dependence, pose challenges for their assessment and control (Van Hout and Norman, 2016; Wells et al., 2018). Since community pharmacies are the most convenient source for individuals to purchase medications for personal use, they can be extremely helpful in promoting responsible use of medicines, which includes alleviating the abuse or misuse and dependence of drugs containing codeine., However, this can only be achieved if community pharmacists are properly trained in harm reduction related to opioids and have strong interprofessional coordination (Mubarak et al., 2023). Thus, this study was designed and conducted with the purpose to ascertain the role of community pharmacists in preventing the misuse or abuse and drug dependence associated with codeine-based analgesic medications in Saudi Arabia.

## 2 Materials and methods

### 2.1 Design of the study

This was a cross-sectional study involving community pharmacists from multiple centers.

### 2.2 Place and duration of study

This study was conducted across 226 community pharmacy outlets located in various cities of Saudi Arabia from 09 Ma y 2023 to 09 October 2023.

### 2.3 Size of the sample

Using a 50% response pattern, 5% margin of error, 99% level of confidence, and a population size of 8,419 licensed community pharmacists (AlRuthia et al., 2018), the minimum effective sample size determined for this study is 368. The sample size needed for this investigation was determined using Raosoft’s sample size calculator (Raosoft, 2020). Three hundred seventy seven community pharmacists participated in the study, however only 226 of them completed all domains of the questionnaire.

### 2.4 Study tool and data collection procedure

The instrument used in this study was a survey questionnaire developed in accordance with the study’s objective and with the assistance of multiple studies and guidelines (ASHP, 2014; RPS, 2021; Algarni et al., 2022). The study tool was reviewed by two research experts, who are affiliated to Johns Hopkins Bloomberg School of Public Health, Maryland, United States, and Ajman University, Ajman, UAE. The reason for the preference was due to their substantial experiences conducting community researches and are well-versed in topics relating to community researches. These factors were therefore taken into account when evaluating the study tool’s face and content validity. In essence, we also took community pharmacists’ opinions into indirect consideration when creating the study tool. This was done based on the results of a comprehensive qualitative study carried out by Alqarni et al., 2022 which took into account a number of themes and subthemes regarding community pharmacists’ opinions and experiences regarding the abuse and misuse of over-the-counter medications. The insightful remarks these experts made were taken into consideration for modifying the study tool. For example, the feedback included suggestions to modify the research title to better align with the study’s aims, to leave the age and years of experience ungrouped instead of grouped, and to improve the wording of certain questions. Following a pilot test of the questionnaire among 20 community pharmacists, a second amendment was made. Most the pharmacists found the ‘multiple choice selection’ options to be ambiguous. Hence, the questionnaire was modified to include a ‘yes/no’ option.

The revised version of the questionnaire contained questions about how pharmacists suspected consumers of abusing or misusing codeine-containing analgesic medications (CCAMs), what strategies they used to combat consumers from abusing, misusing or becoming dependent on CCAMs, and what recommendations they could provid to prevent consumers from abusing, misusing or becoming dependent on CCAMs. Since the participants of the study were community pharmacists with at least a diploma in pharmacy qualification, the English version of the questionnaire was adopted without the need for translation into Arabic language. The questionnaire included the data collector number, demographic characteristics of community pharmacists, community pharmacy-related information, questions on utilization of codeine-containing analgesic medication, and questions focusing on the role and recommendations of community pharmacists in preventing abuse or misuse and dependence of codeine-containing analgesic medications.

The final validated version of questionnaire was incorporated into the REDCap (Research Electronic Data Capture) tools by an expert user from the University of Hail, Saudi Arabia. Study data were collected and managed using REDCap electronic data capture tools hosted at University of Hail, Saudi Arabia (Harris et al., 2009; Harris et al., 2019). REDCap is a safe, web-based software platform that facilitates the collection of data for research studies. In addition to the feature of audit trails for tracking data tampering and export procedures, it has a user-friendly interface for validated data collection.

Twenty data collectors initially agreed to participate and were trained to use the REDCap electronic data capture tool data collection. However, only 14 of them were well-prepared and participated in the data collection process. The data collectors received group training on how to utilize the REDCAP program for data gathering. Indeed, the session or training focused specifically on ethical aspects and data accuracy. Each data collector was given the REDCap questionnaire link to be distributed to the community pharmacies in different cities, particularly major cities of the Kingdom to collect the data. Each data collector was assigned a unique code number together with name of city or area from where they were responsible to collect the data. As the mode of data collection was electronic, study objectives and participants’ written informed consent was included at the beginning of the questionnaire, to ensure that each community pharmacist agreed to proceed with the study questions.

The study included all community pharmacies and pharmacists who were willing to participate, regardless of gender or nationality. However, non-pharmacist personnel, such as support staff, and those who declined to participate were excluded from the study. The community pharmacies in major cities (i.e., Riyadh, Medina, Jeddah, Dammam, Al-Taif, Buraida and Hail) of Saudi Arabia were chosen at random. Data collectors physically visited the pharmacy and briefly described the objective of the study. After the pharmacists agreed to participate, they were provided with the electronic link of the questionnaire prepared and managed by the REDCap electronic data capture tools. The data collectors were directed to wait until the pharmacists were free and willing to entertain, rather than approaching them while they were busy serving customers. Additionally, they were instructed not to become angry or agitated with the pharmacists if they refuse to continue answering the questionnaire or to force them to stop in the middle of the survey if they become preoccupied with entertaining customers.

### 2.5 Analysis of data

The data was extracted from REDCap in the form of the Statistical Package for Social Sciences (SPSS)- The IBM SPSS Statistics, Version: 29.0.0.0 (241) for descriptive and inferential analyses. Given the nominal nature of the data, relationships between individuals’ demographics and pharmacists’ role in preventing abuse or misuse and dependence of CCAMs were examined using Pearson’s chi-squire test/Fisher’s exact test.

This study was reviewed and approved by the Research Ethics Committee at University of Hail, Saudi Arabia dated 23/01/2023, under approval number H-2023-057.

## 3 Results

The majority (75.2%, n = 170) of the community pharmacists were men. More than half (58%, n = 131) fell within the age range of 23–29 years, with a mean age of 31.1 ± 5.196. Saudi pharmacists who graduated from Saudi Universities comprised over half (53.5%, n = 121) of the pharmacists working in the community pharmacies, followed by Egyptian pharmacists who graduated from Egypt (40.7%, n = 92). About one-third (31.9%, n = 72) had working experience of more than 5 years. The majority (60.1%, n = 136) of the community pharmacists handled up to 49 prescriptions daily, and nearly half (47.3%, n = 107) of the pharmacies were situated at a distance of 1 km or more from the nearest hospitals or clinics (Table 1).

**TABLE 1:** Demographic characteristics (n = 226).

Variables		Frequency	Percent
Gender	Male	170	75.2
	Female	56	24.8
Age (years)	23–29	131	58
	30–39	79	35
	40 and above	16	7
Nationality	Saudi	123	54.4
	Non-Saudi	103	45.6
Professional qualification	Master’s degree	14	6.2
	PharmD	106	46.9
	BPharm	97	42.9
	Pharmacy technician (Diploma)	9	4
Country of professional qualification	Saudi Arabia	121	53.5
	Egypt	92	40.7
	Pakistan	5	2.2
	Jordan	5	2.2
	Other	3	1.4
Years of experience in community pharmacy	1 year	48	21.2
	2 years	34	15.1
	3 years	33	14.6
	4 years	22	9.7
	5 years	17	7.5
	>5 years	72	31.9
Number of prescriptions or patients handled in a day	Up to 24	88	38.9
	25–49	48	21.2
	50–74	42	18.6
	75–99	6	2.7
	100 and above	42	18.6
Distance of community pharmacy from the nearest hospital or clinics	Less than 200 m	34	15
	200 to less than 500 m	46	20.4
	500 m to less than 1 km	39	17.3
	1 km or more	107	47.3
Location (city) of community pharmacy	Hail	64	28.3
	Riyadh	63	27.9
	Medina	21	9.3
	Al-Taif	19	8.4
	Jeddah	16	7.1
	Dammam	14	6.2
	Buraidah	8	3.5
	Other	21	9.3

Although the majority (80.5%, n = 182) of the community pharmacists had access to online drug information sources, 19.5% (n = 44) did not have this access. Additionally, nearly half (46.9%, n = 106) of the pharmacists working in community pharmacies did not have a specialized training in substance abuse or misuse.

It was found that the majority (43.8%, n = 99) of CCAM users consumed the medication for 3 days, while 38.1% (n = 86) consumed it for 7 days, 9.8% (n = 22) consumed it for 14 days–28 days, and 8.4% (n = 19) consumed it for more than 28 days. According to the community pharmacists, 55.3% (n = 125) of consumers were aware that using CCAM can lead to addiction. Despite this, if CCAM users missed a dose, 54.9% (n = 124) reported developing a thirst or desire for it.

**FIGURE 1:**
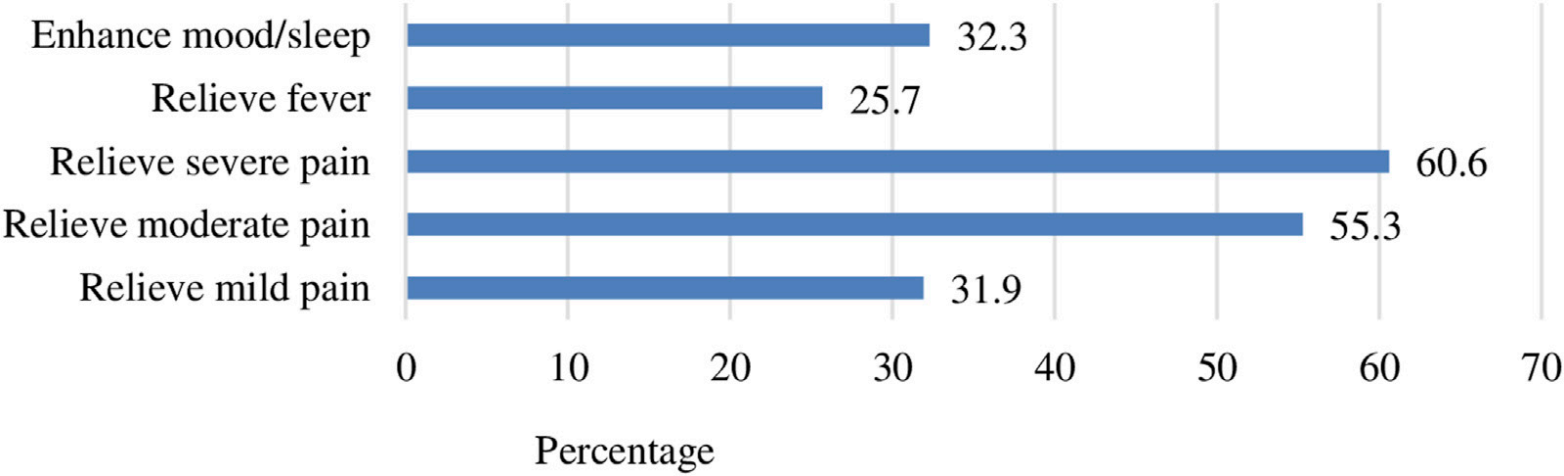
Health conditions prompting customers to purchase CCAMs.

In response to a series of ‘yes/no’ and ‘multiple-choice questions’ regarding the reasons behind consumers’ use of CCAMs, roughly one-third (32.2%) of the pharmacists stated that consumers purchased CCAMs to enhance their mood or sleep, while the majority used them to relieve pain of varying intensities (Figure 1).

**FIGURE 2:**
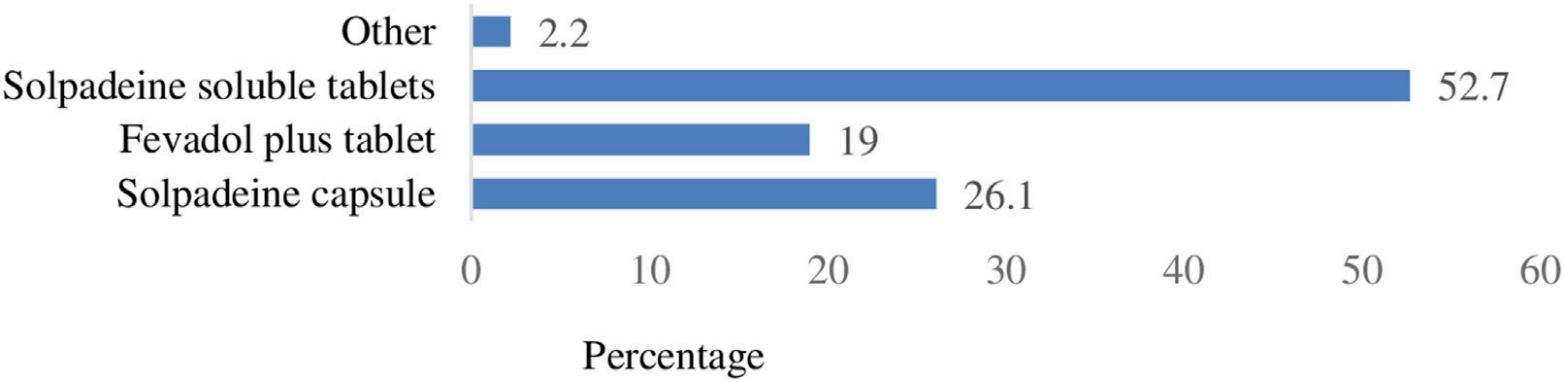
Consumers’ favored CCAM brands.

Consumers’ first choice of CCAM was Solpadeine soluble tablets (53%, n = 119) followed by Solpadeine capsules (26%, n = 59) and Fevadol plus tablets (19%, n = 43) (Figure 2).

**FIGURE 3:**
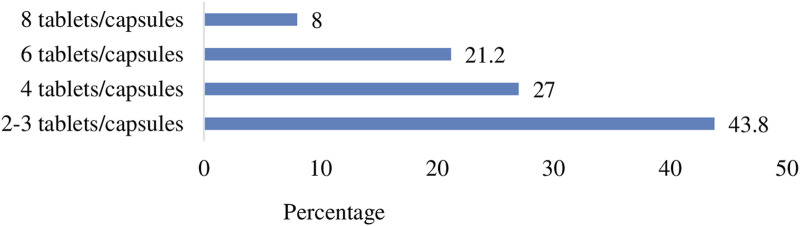
Consumers’ maximum daily dosage of CCAMs.

Although 43.8% (n = 99) of the consumers used to take two to three tablets or capsules daily, a higher proportion of the consumers (56.2%, n = 127) had a tendency to consume four to eight tablets or capsules per day (Figure 3).

Community pharmacists adopted various strategies to identify consumers misusing or abusing CCAMs. Likewise, they brought in practice various approaches, and put forward multiple recommendations towards preventing misuse or abuse and dependence of CCAMs (Table 2).

**TABLE 2:** Community pharmacists’ role towards preventing codeine-containing analgesic medications abuse, misuse, and dependence (n = 226).

Variables	Frequency	Percent
*Signs of suspecting abuse, misuse, and dependence*		
Consumers requesting a large quantity of CCAM at once	200	88.5
Consumers frequently visiting the pharmacy and requesting the same CCAM	187	82.7
Consumers exploiting the crowd to avoid being asked by the pharmacist	160	70.8
Consumers requesting for a specific size of CCAM	150	66.4
Consumers refusing any alternative to CCAM	144	63.7
Consumers visiting the pharmacy during busy time to buy CCAM	117	51.8
*Actions taken by community pharmacists*		
Concealed medicines that could be abused or misused from consumers	198	87.6
Offered an alternative medication when consumers asked for CCAM	183	81
Reduced the dose or quantity of CCAM in the event of suspect of abuse or misuse	147	65
Referred the consumers to physician or primary healthcare center in the event of suspect of abuse or misuse	141	62.4
Refused the sale or deny the availability of CCAM in the event of suspect of abuse or misuse	124	54.9
*Recommendations made by community pharmacists*		
To increase public awareness about the risks of CCAM	192	85
To complete mandatory basic training on addiction and harm reduction	131	58
To monitor prescribed/OTC CCAM	127	56.2
To increase visibility of health warnings on the outside of packs	124	54.9
To develop secure central database to monitor the purchase of CCAM	98	43.4
To prescribing CCAM to be confined to prescribers who are trained in addiction	85	37.6
To health warnings as pop-up feature of online shopping sites	70	31

Herein, we only address the findings that showed statistically significant relationships between individuals’ demographics and pharmacists’ role towards preventing misuse or abuse and dependence of CCAMs. A significant association was noted between refusal to any alternative to CCAM as a suspect of medication abuse or misuse and dependence, and the volume of prescription handled (*p* = 0.041). Similarly, request for a specific size of CCAM, potentially indicating abuse or misuse of the medication, was significantly associated with the age groups (*p* = 0.030).

Reducing the dose or quantity of CCAM in the event of suspect of abuse or misuse was significantly associated with nationality (*p* = 0.005). Similarly, there were significant associations between referring the consumers to physician or primary healthcare center in the event of suspect of abuse or misuse with gender (*p* = 0.004), nationality (*p* < 0.001), and volume of prescription handled (*p* = 0.018). Refusing the sale or denying the availability of CCAM in the event of suspect of abuse or misuse had remarkable association with gender (*p* < 0.001), nationality (*p* < 0.001), and volume of prescription handled (*p* = 0.025).

Recommendation regarding the need of every community pharmacist to complete mandatory basic training on addiction and harm reduction was also perceptibly associated with gender (*p* = 0.008) and nationality (*p* = 0.025). Likewise, recommendation regarding the need of increasing visibility of health warnings on the outside of packs of drugs with abuse or misuse liability had significant association with gender (*p* = 0.037). Recommendation regarding the need of health warnings as pop-up feature of online shopping sites was also significantly associated with gender (*p* = 0.034) and years of work experience (*p* = 0.014).

## 4 Discussion

There is a growing concern regarding the abuse or misuse and dependence on codeine-containing drugs on a global scale. These issues have the potential to impact quality of life, associated costs, and morbidities (Casati et al., 2012; Lo et al., 2015; Khalifeh et al., 2017; Carney et al., 2018; Chan et al., 2021). This necessitates implementation of best practices to ascertain that these medications are used safely and for their intended purpose only. As community pharmacies are among the highly accessible and first point of contact for individuals seeking medical advice and obtaining medications for minor ailments, their role in preventing abuse or misuse and dependence is crucial. This quantitative study, probably the first of its kind in Saudi Arabia, focuses on the role of community pharmacists in preventing the abuse or misuse and dependence of CCAMs. The reason for choosing CCAM lies in the fact that codeine is frequently sold over-the-counter as combination with paracetamol and caffeine.

Our study found that consumers purchased CCAMs to enhance mood or sleep, even though the primary reason for consumption was pain relief. As it is evident that prolonged use of CCAMs can lead to addiction, it is imperative to know whether consumers are aware about their addictive nature. The community pharmacists reported that 55.3% of consumers were aware that using CCAMs can lead to addiction. Conversely, this suggests that a noticeable proportion of customers are still ignorant that using CCAMs may result in addiction. More over half of the consumers developed a craving for it if they missed a dose, which the community pharmacists saw as an indication of addiction based on their interactions and experiences with the consumers. This alarming situation emphasizes the need for collective efforts to raise awareness among consumers. Several studies have reported that codeine-containing preparations are among the most frequently abused or misused over-the-counter medications, particularly among younger individuals (Wright et al., 2016). The accessibility of codeine-based medicines is one of the factors contributing to their increased misuse in countries where they are available over-the-counter further exacerbating their addictive nature (Robinson et al., 2010; Nielsen and Van Hout, 2017; Van Hout et al., 2018; Wells et al., 2018).

Over 56% of the consumers consumed four to eight tablets or capsules of CCAMs per day. This suggests a lack of public awareness regarding the “risks and harms associated with higher doses and long-term use” of CCAMs. Furthermore, each Solpadeine soluble (effervescent) tablet contains paracetamol 500 mg, codeine 8 mg, and caffeine 30 mg. The inclusion of caffeine (a central nervous system stimulant) in the formulation and faster onset of action due to effervescent nature of the preparation might be the reason for the consumers’ preference to this brand despite the availability of Fevadol plus tablets, which are comparatively cheaper than Solpadeine effervescent tablets (SFDA, 2024).

The community pharmacists were asked a set of diverse questions related to the role they play in preventing CCAMs abuse, misuse and dependence. Community pharmacists assessed the patients through observation of their behavior and attitude. Although varied responses were observed from the pharmacists regarding each question or situation regarding identifying the users, behaviors such as requesting for larger quantity, frequently visiting the pharmacy, and requesting for the same brand of CCAM, visiting the pharmacy during busy time, refusing any alternative to CCAM, exploiting the crowd to avoid being asked by the pharmacist, and requesting for a specific size were indicative of identifying CCAM users. The study by Carney et al., 2016 in the United Kingdom also found similar behaviors suggestive of users (Carney et al., 2016).

Community pharmacists play a vital role in resolving the issue of CCAMs abuse most frequently through keeping medicines with abuse or misuse potential out of consumers’ sight and offering an alternative medication, followed by reducing the dose or quantity, referring the consumers to physician or primary healthcare centers. The above mentioned roles of community pharmacists in preventing the abuse or misuse of CCAM has been highlighted in several studies (Bach and Hartung, 2019; Ylä-Rautio et al., 2020; Algarni et al., 2022). However, the act of refusing to sell or denying the availability of CCAM was not as common in practice. This is particularly due to safety concerns, as users often argue and sometimes become violent when faced with denial or referral. However, they could just as easily get the medicine by going to another pharmacy. Consequently, the pharmacists may hesitate to refuse the sale out of fear of losing customers. This observation is supported by (Sansgiry et al., 2017).

In this study, community pharmacists’ experiences led them to identify a number of recommendations that could play pivotal role in reducing abuse, misuse and dependence of CCAMs. Increasing public awareness about the risks of CCAMs was among the most common recommendations made by the pharmacists. Studies have also shown that increasing public awareness and proper counseling could assist in preventing CCAM abuse, misuse, and dependence (Bergin et al., 2015; Norman et al., 2016; Zaprutko et al., 2016; Hill et al., 2018; Mody et al., 2020). Mandatory basic training on addiction and harm reduction, monitoring of prescribed or over-the-counter CCAMs, and increasing the visibility of health warnings on the outside of packs were among the other recommendations made. Community pharmacists also highlighted the need of developing secure central database to monitor the purchase of CCAMs, prescribing CCAMs to be confined to prescribers who are trained in addiction, and implementing health warnings as pop-up alerts on online shopping platforms to prevent abuse, misuse, and dependence.

The authors propose the following implications for policy, practice, and future research based on the study’s findings.

• Addressing the differences between knowledge and practice regarding the appropriate use of CCAMs is imperative in order to prevent their abuse, overuse, and dependence through coordinated monitoring.
• Enhancing a pharmacist’s proficiency in these domains may help ensure the safe and efficient administration of opioids, improve the general health of the country, aid in ending the cycle of substance abuse and dependency, and prevent young people from forming unhealthy habits. The future of the country would ultimately gain a great deal from having young people free from addiction.
• The promotion of improved patient counseling regarding CCAMs’ hazards have to get special attention.
• As a requirement for employment, community pharmacists must complete a necessary specialized course on substance abuse and have access to online drug information (DI) sources.
• The subject of substance use, along with methods to reduce abuse, misuse, and dependence, must be thoroughly covered in the undergraduate curriculum. The effects of substance abuse, misuse, and dependence on the general health, and future of the country should also be emphasized.
• More research is needed to develop collaborative practice models, evidence-based opioid stewardship interventions, and clinical support systems that facilitate better communication between prescribers, pharmacists, and regulatory agencies.
• The study’s findings may help guide policymakers who want to increase community pharmacists’ contribution in reducing drug addiction, misuse, and dependence across the country.

The absence of comprehensive information regarding community pharmacies and community pharmacists prevented this study from selecting participants using a specified random sampling approach, rather than simple random. Although the approach was to recruit community pharmacists working in urban, semi-urban and rural areas of the country, we could not enroll the pharmacists from all the regions except the major cities. Additionally, we could succeed to enroll pharmacists from only some pharmacy chains despite our maximal efforts. Moreover, the response rate was also low. Hence, study findings may not be generalized. However, the exploratory nature of this study still offers key insights into understanding the role of community pharmacists in dealing with the public health issue of CCAMs abuse, misuse, and dependence despite these limitations. These limitations could be addressed in future research.

## 5 Conclusion

While changing the behaviors of users is a challenging task, community pharmacists, as the first point of contact and being more accessible, could play an important role in preventing abuse or misuse and dependence on codeine-containing analgesic medications. Therefore, it is essential to reinforce their roles and mandate their training on substance use and abuse. This also draws attention to the need of a multifaceted collective approach in the manufacturing, advertising, prescribing, and dispensing of medications with abuse potential. Government regulatory agencies should develop comprehensive plans to raise awareness and enforce stricter regulatory controls on the safe and effective use of codeine-based drugs. This could potentially reduce codeine-associated morbidity and mortality, improve quality of life, and save national resources.

## Data Availability

The datasets presented in this study can be found in online repositories. The names of the repository/repositories and accession number(s) can be found in the article/supplementary material.
